# Improved tactile speech perception using audio-to-tactile sensory substitution with formant frequency focusing

**DOI:** 10.1038/s41598-024-55429-3

**Published:** 2024-02-28

**Authors:** Mark D. Fletcher, Esma Akis, Carl A. Verschuur, Samuel W. Perry

**Affiliations:** 1 University of Southampton Auditory Implant Service, University of Southampton, University Road, Southampton, SO17 1BJ UK; 2 Institute of Sound and Vibration Research, University of Southampton, University Road, Southampton, SO17 1BJ UK

**Keywords:** Translational research, Auditory system, Sensory processing, Somatosensory system

## Abstract

Haptic hearing aids, which provide speech information through tactile stimulation, could substantially improve outcomes for both cochlear implant users and for those unable to access cochlear implants. Recent advances in wide-band haptic actuator technology have made new audio-to-tactile conversion strategies viable for wearable devices. One such strategy filters the audio into eight frequency bands, which are evenly distributed across the speech frequency range. The amplitude envelopes from the eight bands modulate the amplitudes of eight low-frequency tones, which are delivered through vibration to a single site on the wrist. This tactile vocoder strategy effectively transfers some phonemic information, but vowels and obstruent consonants are poorly portrayed. In 20 participants with normal touch perception, we tested (1) whether focusing the audio filters of the tactile vocoder more densely around the first and second formant frequencies improved tactile vowel discrimination, and (2) whether focusing filters at mid-to-high frequencies improved obstruent consonant discrimination. The obstruent-focused approach was found to be ineffective. However, the formant-focused approach improved vowel discrimination by 8%, without changing overall consonant discrimination. The formant-focused tactile vocoder strategy, which can readily be implemented in real time on a compact device, could substantially improve speech perception for haptic hearing aid users.

## Introduction

Sensory substitution devices that convert audio into tactile stimulation were used in the 1980s and early 1990s to support speech perception in people with a severe or profound hearing loss. These haptic hearing aids (also called “tactile aids”) allowed users to learn a large vocabulary of words through tactile stimulation alone^1^ and could substantially improve word recognition with lip reading^2–4^. However, by the mid-to-late 1990s, haptic hearing aids were rarely used clinically because of large improvements in the effectiveness of cochlear implants (CIs)^5^ and critical limitations in the haptic technology available^5,6^. While CIs have been life-changing for hundreds of thousands of people, millions in low-resource settings still cannot access them because of their high cost and the need for advanced healthcare infrastructure^7^. Even in high-resource settings, many are unable to access CIs because of barriers in complex care pathways^8^ and because of disorders that prevent implantation (such as cochlear ossification). Furthermore, while CIs often effectively restore speech recognition in quiet listening environments, users typically have substantial difficulties understanding speech in background noise^9,10^ and locating sounds^11^. A new generation of haptic hearing aids that exploit the huge recent advances in compact haptic actuator, battery, and microprocessor technology might now be able to offer a viable low-cost, non-invasive, and highly accessible alternative or complement to the CI.

Previously, many haptic hearing aids have transferred audio frequency information by mapping different frequencies to different locations of tactile stimulation on the skin^12–16^. Now, cutting-edge wide-band haptic actuator technology allows new audio-to-tactile conversion strategies, with a frequency-to-frequency mapping, to be deployed on wearable devices. One such strategy is the tactile vocoder^9–11,17–19^. In this approach, audio is first filtered into different frequency bands. The amplitude envelope is extracted from each of these bands and used to modulate the amplitude of low-frequency vibro-tactile tones. The number of tactile tones typically matches the number of frequency bands, with each band modulating a different tone. This approach allows the frequency range of speech to be converted to the frequency range where tactile sensitivity is high. The tactile tones are presented through vibro-tactile stimulation at a single site.

The frequency-to-frequency tactile vocoder strategy has been successfully used to improve speech-in-noise performance^9,10,17,20^ and sound localisation^11,19^ for CI users with accompanying audio (“electro-haptic stimulation”^9^) and to transfer speech information without accompanying audio^18^. However, while the latest iteration of the tactile vocoder strategy can effectively transfer some important phonemic information, such as that used for discrimination of voiced and voiceless consonants, it is poor at transferring phonemic cues for vowels and obstruent consonants^18^. Obstruent consonants are formed by obstructing airflow and include plosives (such as /*p*/), which are generated via closure followed by an abrupt release, and fricatives (such as /*f*/), which are generated via airflow through a narrow opening in the vocal tract.

The latest tactile vocoder strategy distributes audio frequency bands across the speech frequency range using a rule that mimics the healthy auditory system (though with a much lower resolution; see “Methods”)^9,17,18,20^. In the current study, we tested two alternatives to this “wide focused” filtering approach. The first “formant focused” approach aimed to improve vowel discrimination by focusing more bands around the first and second formant frequencies (300–2500 Hz). The second “obstruent focused” approach aimed to improve obstruent consonant discrimination by more densely focusing bands at higher speech frequencies (2500–7000 Hz). These new approaches exploit the fact that the tactile system does not make assumptions about how speech will be distributed across frequency (because speech is not usually received through vibration). In contrast, the auditory system does have an expectation of how speech will be distributed across frequency, which can be disrupted when frequency information is warped to focus on specific speech features^21,22^.

**Figure 1:**
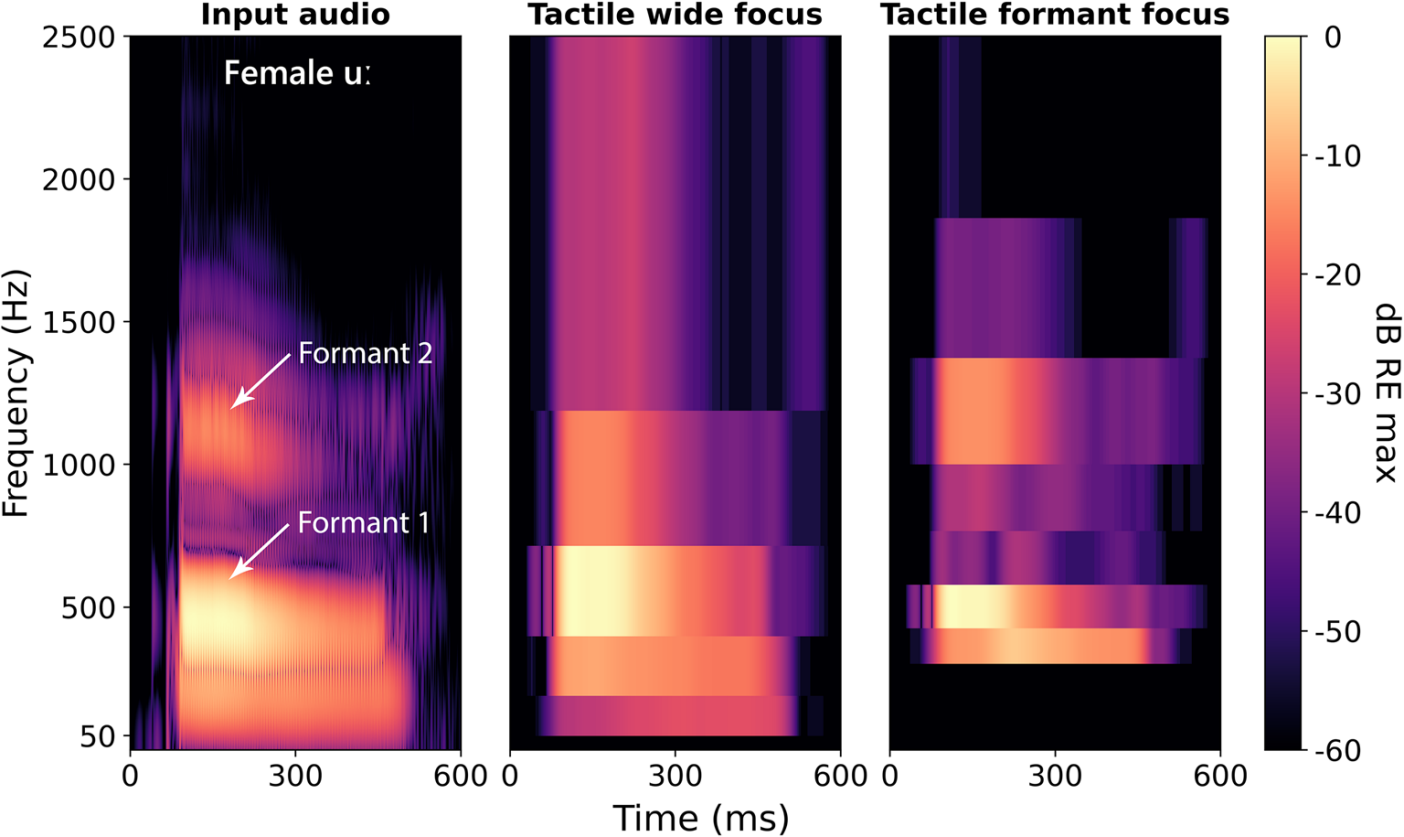
Spectrograms for the vowel /uː/ (as in “blue”) spoken by the female talker from the EHS Research Group Phoneme Corpus (see “Methods”). The left panel shows the input audio, and the central and right panels show the tactile envelopes extracted using the wide-focused (baseline) and the newly developed formant-focused vocoder strategies used in the current study. The frequency range shown focuses on the lower frequencies around the first and second formants, which are marked for the input audio. The audio spectrogram sample rate was 22.05 kHz, with a window size of 8 ms (Hann) and a hop size of 1 sample. Each window was zero-padded to a length of 8192 samples. The tactile spectrogram sample rate was 16 kHz (matching that used in the current study), with no windowing applied. For the input audio, intensity is shown in decibels relative to the maximum magnitude of the short-time Fourier transform. For the tactile envelopes, intensity is shown in decibels relative to the maximum envelope amplitude. The spectrograms were generated using the Librosa Python library (version 0.10.0).

Figure 1 shows an example of how the formant-focused approach can more effectively extract the first and second formants than the wide-focused approach, for the vowel /*uː*/. With wide focusing (central panel), the two formants are not well distinguished, with a single broad lower-frequency peak in energy portrayed. In contrast, with formant focusing (right panel), the two formants are clearly distinguishable. Formants are critical to vowel perception and so this better formant representation was expected to improve vowel discrimination.

The effect of formant focusing on consonant perception was anticipated to be more complex, as the importance of formants differs substantially across consonant types. Improved discrimination would be expected for sonorant consonant pairs (approximants, such as /*w*/, which are generated via formant resonances in a partially closed vocal tract, and nasals, such as /*n*/, which are generated by transmission through the nasal cavity) that differ by manner and place of articulation, as the frequency and amplitude of the second formant is important in these distinctions. In contrast, the focusing of frequency bands towards lower formant frequencies might worsen performance for consonants that rely on gross spectral shape at higher frequencies (e.g., fricatives or plosives). Performance might also be reduced for contrasts that rely on the distinction between voiced and voiceless cognates (phonemes produced via the same manner and place of articulation and differing only by whether they are voiced), because of the lack of a frequency band at the voicing bar (around the fundamental frequency of a talker’s voice). However, note that previous work in hearing has shown that voicing perception can be tolerant to the removal of lower frequency audio information^23,24^. Because of the hypothesised both positive and negative impacts of formant focusing, it was anticipated that overall performance with consonants would be unaltered.

**Figure 2:**
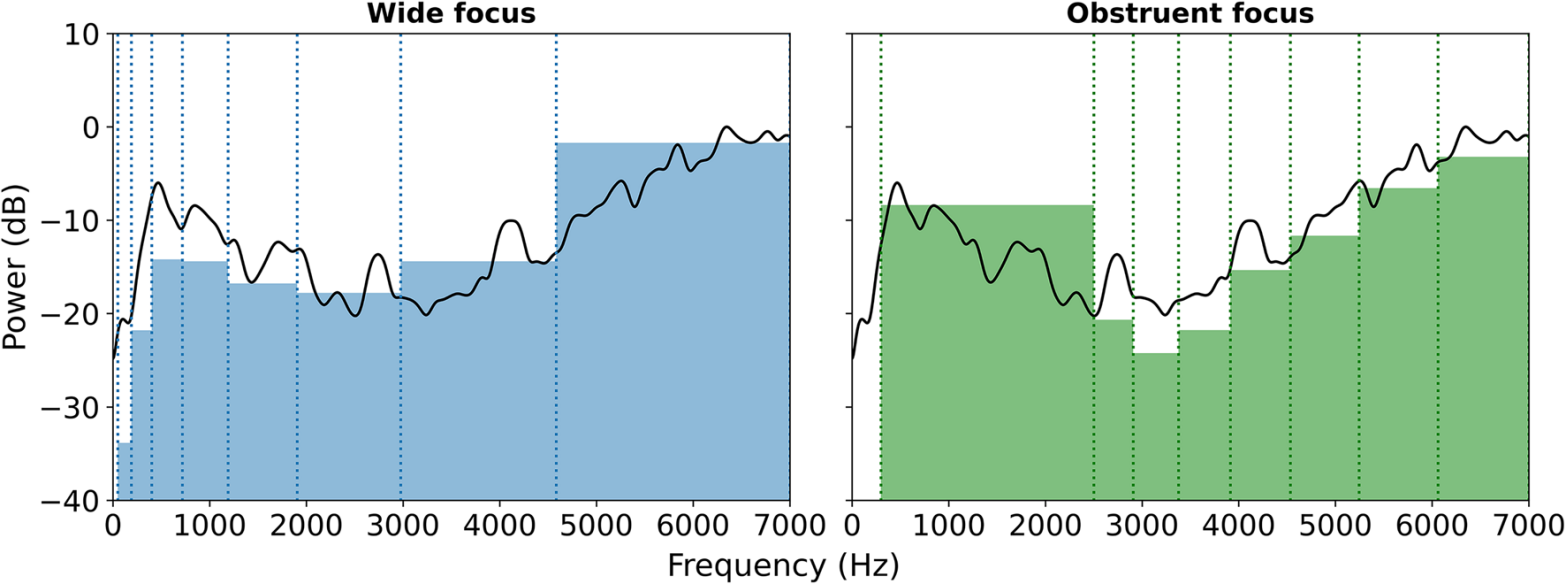
The frequency spectrum for the consonant /s/ (spoken by the male talker), with wide focusing (left) and obstruent focusing (right). The plot shows the audio spectrum (black line) and the average envelope amplitude in each frequency band (with the band limits highlighted using dashed lines). Spectrums were generated by calculating the power spectral density (PSD) of the original audio, using a window length of 256 samples and an overlap of 128 samples. The windows were zero-padded to a length of 8192 samples. The envelope amplitudes were extracted using the wide and obstruent focused approaches used in the current study (see “Methods”). The envelopes were normalised by subtracting the difference between the average envelope amplitude, weighted by the width of each frequency band, and the average amplitude of the PSD.

The aim of obstruent focusing was to better represent mid-to-high frequency noise components (bursts and frication noise) and thereby improve discriminability of obstruent consonants. An example of this can be seen in Fig. 2, which shows the spectral representation for the consonant /s/, with wide and obstruent focusing. Obstruent focusing dedicates more bands to the upwards spectral tilt at mid-to-high frequencies than wide focusing, with the tilt coded by the highest six frequency bands for obstruent focusing and only the highest three bands for wide focusing. Spectral characteristics such as tilt are important for obstruent phoneme perception^25^. While obstruent focusing was expected to improve performance for plosives and fricatives, it was anticipated to reduce performance for voiced-voiceless contrasts as so few frequency bands were focused near the voicing bar. Obstruent focusing was also expected to have a small negative effect on vowel discrimination. While the first and second formants, which are critical to vowel perception, are poorly represented with obstruent focusing, this was expected to be partially compensated for by better representation of the higher-frequency third and fourth formants.

## Results

**Figure 3:**
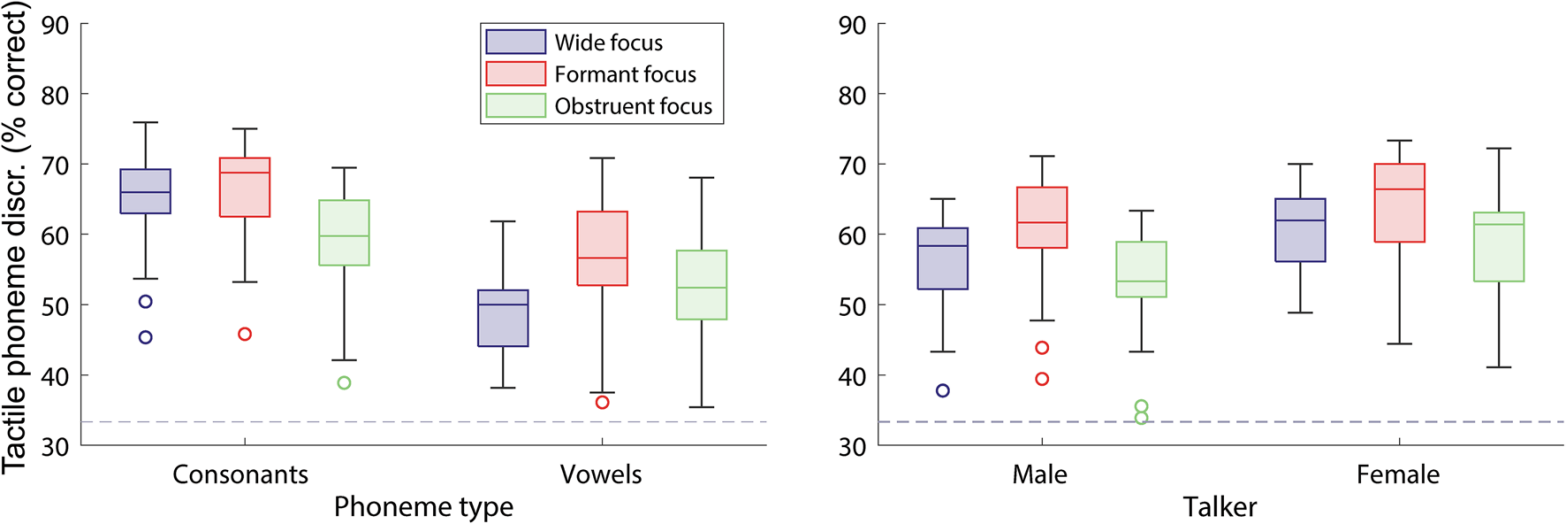
Percentage of phoneme pairs discriminated for each focusing approach, with either the different phoneme types (consonants or vowels; left panel) or different talkers (male or female; right panel) shown separately (N = 20). The horizontal line inside the box shows the median, and the top and bottom edges of the box show the upper (0.75) and lower (0.25) quartiles. Outliers (values of more than 1.5 times the interquartile range) are shown as unfilled circles. The whiskers connect the upper and lower quartiles to the maximum and minimum non-outlier values. Chance performance is marked by a dashed grey line.

Figure 3 shows the percentage of phonemes discriminated with the three focusing approaches, for the 20 participants who took part in this study. Results are shown either for each phoneme type (left panel) or each talker (right panel). A three-way repeated-measures analysis of variance (RM-ANOVA) was run with the factors: focusing approach (wide, formant, or obstruent focused), phoneme type (consonants or vowels), and talker (male or female). Main effects were found for the focusing approach (*F*(2,38) = 25.5, *p* < 0.001; partial eta squared (η^2^) = 0.573), phoneme type (*F*(1,19) = 150.1, *p* < 0.001; η^2^ = 0.888), and talker (*F*(1,19) = 39.8, *p* < 0.001; η^2^ = 0.677). No interaction was found between talker and either phoneme type (*F*(1,19) = 1.6, *p* = 0.223) or focusing approach (*F*(2,38) = 2.2, *p* = 0.129), or between talker, phoneme type, and focusing approach (*F*(2,38) = 0.5, *p* = 0.608). A significant interaction was found between focusing approach and phoneme type (*F*(2,38) = 19.1, *p* < 0.001; η^2^ = 0.501).

Overall performance with wide focusing was 58.2% (standard deviation (SD): 6.4%), with formant focusing was 62.2% (SD: 8.0%), and with obstruent focusing was 56.0% (SD: 7.8%). With wide focusing, performance was 15.9% higher for consonants than for vowels (SD: 6.9%); with formant focusing, performance was 9.6% higher (SD: 4.3%); and, with obstruent focusing, performance was 5.8% higher (SD: 5.8%). Performance with the female talker was higher for wide focusing by 4.8% (SD: 4.4%), for formant focusing by 3.7% (SD: 4.9%), and for obstruent focusing by 5.9% (SD: 3.8%).

Contrasts revealed a significant overall improvement in performance with formant focusing compared to the wide-focusing baseline (*F*(1,19) = 27.5, *p* < 0.001; η^2^ = 0.591). Formant focusing improved performance across all phonemes by 3.9% on average (ranging from − 4.7 to 10.3%; SD: 4.5%). The size of this improvement was significantly larger for vowels than for consonants (*F*(1,19) = 13.2, *p* = 0.002; η^2^ = 0.409). For vowels, performance with formant focusing was 7.7% higher on average than with wide focusing (ranging from − 4.9 to 18.8%; SD: 7.0%) and, for consonants, was 1.4% higher on average (ranging from − 4.6 to 7.9%; SD: 3.3%). The overall benefit of formant focusing compared to wide focusing was not found to depend on the talker (*F*(1,19) = 1.0, *p* = 0.335).

Contrasts showed no significant overall difference in performance with obstruent focusing compared to wide focusing (*F*(1,19) = 1.6, *p* = 0.218). However, the effect of obstruent focusing compared to wide focusing was found to significantly differ between consonants and vowels (*F*(1,19) = 38.9, *p* < 0.001; η^2^ = 0.672). For consonants, performance with obstruent focusing was 6.3% lower on average than with wide focusing (with reductions ranging from 0.0 to 13.4%; SD: 3.0%) and, for vowels, performance was 1.4% higher on average (ranging from − 10.4 to 16.0%; SD: 7.3%). The overall difference between obstruent focusing and wide focusing was not found to depend on the talker (*F*(1,19) = 1.3, *p* = 0.266).

Planned *post hoc t*-tests (corrected for multiple comparisons; see “Methods”) were run to compare formant focusing to obstruent focusing. Across all phonemes, performance was 6.2% better with formant focusing (ranging from 0.0 to 11.1%; SD: 3.3; *t*(19) = 8.7, *p* < 0.001; Cohen’s *d* = 0.76). For consonants, formant focusing was 7.7% better (ranging from 0.9% to 14.4%; SD: 4.3%; *t*(19) = 8.0, *p* < 0.001; *d* = *0.94*), and for vowels formant focusing was 3.9% better (ranging from − 6.9% to 15.3%; SD: 5.3%; *t*(19) = 3.2, *p* = 0.004; *d* = 0.44).

**Figure 4:**
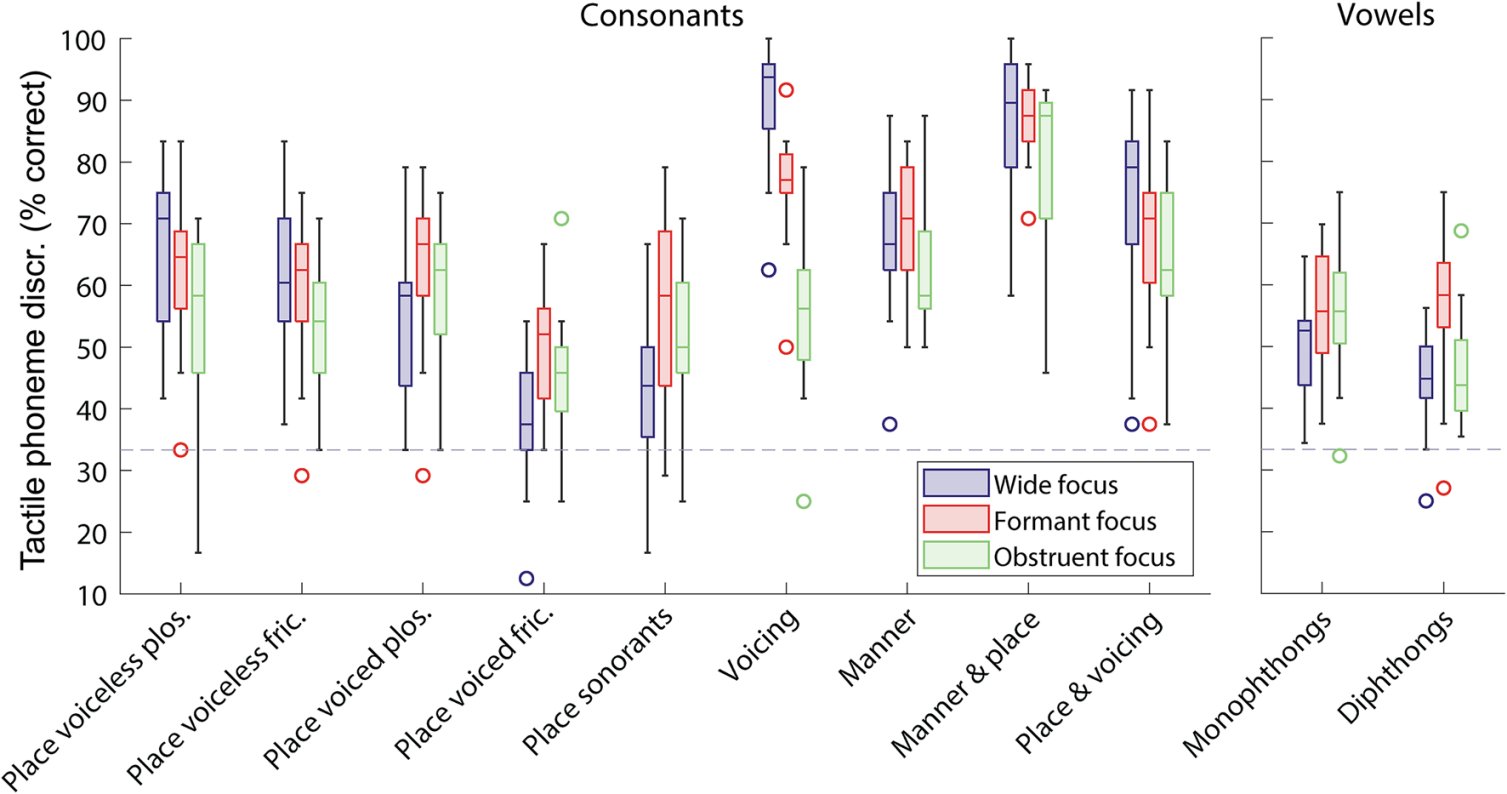
Percentage of phonemes discriminated for the different focusing approaches, grouped by phoneme contrast type (N = 20). Box plots are shown as in Fig. 3. Chance performance is marked with a dashed grey line.

Figure 4 shows phoneme discrimination for each phoneme subgroup. Further *post hoc* analyses (corrected for multiple comparisons) revealed that phoneme discrimination was significantly better with formant focusing than with wide focusing in some subgroups. For voiced fricatives and for sonorants that differed by place of articulation, performance improved with formant focusing by 11.5% (SD: 10.5%; *t*(19) = 4.9, *p* = 0.002) and 13.8% (SD: 12.5%; *t*(19) = 4.9, *p* = 0.002), respectively. Improvement in performance for voiced plosives differing by place of articulation was also close to significance (mean change in performance of 8.3%; SD: 11.4%; *t*(19) = 3.3, *p* = 0.057). Performance decreased for phoneme pairs differing by whether they were voiced or voiceless by 13.3% (SD: 11.0%; *t*(19) = 5.4, *p* < 0.001). For vowels, formant focusing improved performance for monophthongs by 5.8% (SD: 7.1%; *t*(19) = 3.7, *p* = 0.026) and for diphthongs by 11.5% (SD: 13.4%; *t*(19) = 3.8, *p* = 0.020).

Changes in performance for phoneme sub-groups were also observed for obstruent focusing compared to wide focusing. No significant improvement in performance with obstruent focusing was observed for any consonant subgroup, although improvement for sonorants that differ by place of articulation approached significance (mean change in performance of 8.5%; SD: 12.6%; *t*(19) = 3.0, *p* = 0.077). Performance worsened with obstruent focusing compared to wide focusing by 34.8% for consonants differing by whether they were voiced or voiceless (SD: 10.4%; *t*(19) = 14.9, *p* < 0.001), by 11.5% for voiceless plosives differing by place of articulation (SD: 11.5%; *t*(19) = 4.4, *p* = 0.005), and by 6.9% for consonants differing by both manner and place of articulation (SD: 7.6%; *t*(19) = 4.1, *p* = 0.012). Decreased performance was also close to significance for voiceless fricatives differing by place of articulation (mean decrease of 7.9%; SD: 11.1%; *t*(19) = 3.2, *p* = 0.056) and for consonants differing by both place of articulation and voicing (mean decrease of 10.6%; SD: 14.6%; *t*(19) = 3.3, *p* = 0.058). No significant change for vowel subgroups was observed, although improvement in performance approached significance for monophthongs (mean improvement of 5.2%; SD: 7.7%; *t*(19) = 3.0, *p* = 0.077).

Additional exploratory analyses assessed whether there was a correlation between phoneme discrimination (for wide, formant, or obstruent focusing approaches) and either age or detection thresholds for a 125-Hz vibro-tactile tone (measured during screening). No evidence of a correlation between phoneme discrimination and either age or detection threshold was found.

Finally, to assess whether fatigue, training, or adaptation effects might have influenced the outcomes, performance was assessed for each of the four repeat measurements made with each phoneme pair and focusing approach. Note that each of these four repeats was completed in sequence so that, for example, all phoneme pairs and focusing approaches were measured once before any of the second repeat measurements were made. For each repeat, the order of conditions was re-randomised. For the first repeat, the mean performance across all phoneme pairs and focusing approaches was 59.4% (SD: 7.2%), for the second repeat was 59.6% (SD: 8.0%), for the third repeat was 57.9% (SD: 6.9%), and for the final repeat was 58.0% (SD: 8.2%).

## Discussion

Previously, it has been shown that tactile phoneme discrimination with the latest wide-focused tactile vocoder strategy is good for consonants but poor for vowels^18^. The current study tested a new version of the strategy, which was designed to improve vowel discrimination by better transferring formant information. As expected, vowel discrimination was substantially improved with this new formant-focused approach, while overall consonant discrimination remained unaffected. In addition to being critical for haptic hearing aids that target those unable to access CIs, enhanced vowel perception could be crucial for augmenting CI listening, particularly for lower-performing users who tend to have poor vowel perception even in quiet listening conditions^26^.

While the formant-focused vocoder strategy did not affect overall consonant performance, it improved discrimination for some consonant sub-groups and worsened discrimination for others. Improved discrimination was observed for voiced sonorants. This may have been due to better representation of the second formant, which is important for place contrasts among nasals or approximants. Unexpectedly, an improvement in performance was also observed for voiced fricatives that differ by place of articulation. Voiced fricatives have a “dual spectrum”, with a low-frequency component at the voicing bar generated by the vocal folds, and a high-frequency noise component generated by turbulent airflow in the oral cavity. Formant focusing might have increased separation of these components across the vibro-tactile tones through the denser concentration of mid-frequency bands, making them more salient. Additionally, the spectral tilt of the mid-to-high frequency portion of the noise component may have been portrayed more effectively.

Discrimination of pairs differing by manner and place of articulation did not improve with formant focusing, contrary to our expectation. This may have been due to the second formant being relatively weak and close in frequency to the first formant for these phonemes. Even with formant focusing, there may not have been adequate frequency separation or dynamic range available to sufficiently represent the second formant.

Formant focusing worsened performance for contrasts between voiced and voiceless consonants. This was expected as the two frequency bands that were focused on the voicing bar with the wide-focused approach were reallocated to formant frequencies. A future iteration of the formant-focused approach might explore whether allocating one or more of the bands to the voicing bar can recover discrimination of consonants differing by voicing, without reducing the benefits of formant focusing. Voicing information is not accessible through lip reading and so effectively transferring this information could be particularly important for those who receive limited acoustic information through other means (e.g., their CI)^27^. Indeed, improved voicing perception has already been identified as an important benefit of bimodal stimulation, where CI listening is supplemented by residual low-frequency acoustic hearing, in the small percentage of CI users for whom this is possible^28^.

In addition to the formant-focused approach, another new approach was tested that concentrated frequency bands towards higher speech frequencies to improve obstruent consonant discrimination. This approach was found to be ineffective. In fact, overall discrimination of consonants was worse with obstruent focusing than with the original wide-focused approach. This may in part reflect the greater importance of representing lower formants for sonorant (approximants and nasal) consonants. As expected, performance on consonants differing only by voicing was substantially impaired with obstruent focusing. This was likely because frequency bands focused on or close to the voicing bar were reallocated to higher frequencies (no bands represented frequencies below 300 Hz and only one band represented frequencies between 300 and 2500 Hz). For vowels, the expected reduction in performance with obstruent focusing compared to wide focusing was not observed. This was likely due, at least in part, to the increased resolution at higher speech frequencies improving the representation of the higher formants, which can be used for vowel discrimination^29^.

Overall performance, across all focusing approaches, was found to be better for the female than for the male talker. This may have been partly due to spectral factors, such as the wider frequency spacing of formants for the female talker and the good alignment of the formants with the tactile vocoder filter bands (as shown in Fig. 1). Differences in broadband amplitude modulation profiles between the talkers^30^ may also have played an important role. This is supported by a previous study of tactile phoneme discrimination with the same talkers, which found better performance with the female talker when only broadband amplitude envelope cues were presented, precluding the influence of spectral cues^18^.

In the current study, training was deemed unnecessary because of the simplicity of the phoneme discrimination task. It was shown that, despite performance feedback being given on each trial (which would aid learning), scores were highly stable across different time points in the testing session (which lasted approximately two hours in total). In addition to indicating that training effects were minimal, this suggests that factors such as fatigue and long-term adaptation (e.g.,^31^) also had little or no impact. The absence of a requirement for training presents a significant advantage, as it allows relatively rapid testing of alternative audio-to-tactile conversion strategies.

The lack of a need for training also stems from limitations of the phoneme discrimination task. In higher-level tasks involving words or sentences, significant improvements with training have been observed for tactile-only speech in quiet^1^, for tactile stimulation used to support lip reading^32^, and for audio-tactile speech in noise with CI users^9^ or with simulated CI audio in normal-hearing listeners^17,33^. The phoneme discrimination task concentrates on spectral or spectral-temporal aspects of speech, and not on detection of the temporal boundaries of words, syllables, or phonemes in running speech (segmentation), which is important in higher-level tasks. Previous studies have shown evidence that important segmentation cues can be effectively delivered by providing syllable timing cues using tactile pulses^34^ or by using tactile stimulation derived from the broadband amplitude envelope^35^. The wide-focused tactile vocoder strategy has previously been shown to substantially improve phoneme discrimination compared to the broadband amplitude envelope^18^, and the formant-focused tactile vocoder has been shown in the current study to further improve discrimination. This would be expected to facilitate better segmentation by making phoneme distinctions clearer^36^. However, the relationship between tactile phoneme discrimination and speech segmentation is not yet well understood. Future work is required to confirm that the benefits of formant focusing shown in the current study translate to benefits in more realistic speech testing conditions.

Another limitation of the current study is that the participant demographic did not match the target user group for haptic hearing aids. All participants were under 40 years of age, but a substantial portion of people with hearing loss are older. No evidence of a correlation between age (which spanned 13 years) and tactile phoneme discrimination ability was found in the current study or in previous work using the tactile vocoder^18^. Previous studies showing speech-in-noise performance for CI users can be improved with tactile stimulation have also found no evidence of a relationship between age and tactile benefit^9,10,17,20^. While aging does not appear to affect tactile intensity discrimination^37,38^ or temporal gap detection for vibro-tactile tones^39^, vibro-tactile detection thresholds^40,41^ and frequency discrimination^42^ are both known to worsen with age. This reduced tactile dynamic range and frequency resolution would be expected to decrease the amount of speech information transferred using the tactile vocoder strategy. However, the current study and previous work found no relationship between vibro-tactile detection threshold and either tactile phoneme discrimination performance^18^ or audio-tactile benefit^9,10,17,20^. Nonetheless, in future work it will be important to establish what speech information can be effectively extracted from tactile stimulation in different user groups.

As well as not fully spanning the age range of the target user group, participants in the current study reported having no hearing impairment. Several studies have found no differences in tactile speech performance between normal-hearing and hearing-impaired individuals^9,17,18,43,44^. For example, similar improvements in speech-in-noise performance with tactile stimulation using the tactile vocoder strategy were observed for CI users and for normal-hearing individuals listening to CI simulated audio^9,10,17^. However, there is evidence of increased tactile sensitivity in congenitally deaf individuals^45^, and the current study might therefore underestimate performance for this group. Further work is needed to conclusively determine whether tactile speech perception differs between normal-hearing listeners and those with hearing loss.

Future studies should also explore whether additional sound information can be transferred by extending the formant-focused tactile vocoder strategy so that it uses multiple tactile stimulation sites. Studies with arrays of actuators have shown that vibrations are localised more precisely around the wrist than along the forearm^46,47^ and that at least four actuators distributed around the wrist can be accurately discriminated^48,49^. However, this does not consider practical challenges that would be faced when building a device for the real world. For example, microchips, batteries, and buckle mechanisms limit where actuators can be placed, and actuators at the palmar wrist can become audible and change their response characteristics if the user couples them with a surface, as is common in everyday activities like cooking or typing at a keyboard^50^. The use of additional stimulation sites might allow the delivery of phoneme information that was not optimally transferred with formant focusing, such as low-frequency voicing or pitch cues (e.g.^12^). It could also allow transfer of additional high-frequency sound information, which is important for sound localisation with haptics^19^. In previous haptic sound-localisation studies, spatial hearing cues have been effectively delivered through differences in stimulation across the wrists^11,19,37,50^, which leaves open the possibility of transferring additional information through more local changes in stimulation around the wrists. Alternatively, multiple sites might be used to increase the tactile dynamic range available by transferring additional intensity information through the perceived spread of stimulation across nearby sites.

Another important area for future work will be establishing and maximising the robustness of the formant-focused vocoder strategy to background noise. CI users often struggle to identify vowels in background noise^51^, and so a noise-robust version of this new strategy could yield larger benefits of tactile stimulation to speech-in-noise performance than previous tactile vocoder methods^9,10,17^. Recent studies suggest that amplitude envelope expansion, which exaggerates larger amplitude envelope fluctuations, improves the noise-robustness of the tactile vocoder^9,10,17^ and that high-frequency sound information can be critical for separating speech and noise sources coming from different locations^10^. Further investigation of the importance of dedicating bands to higher frequencies and of envelope expansion methods for improving noise robustness is required. In addition, the effectiveness of traditional noise-reduction methods, such as minimum mean-square error estimators^52^, and of more advanced techniques, like those exploiting neural networks^53^, should be assessed for tactile speech in noise.

Whether the effectiveness of haptic hearing aids can be improved by adapting the stimulation strategy to the individual user should also be explored. For example, the dynamic range of the device could be adapted based on the user’s detection thresholds, as is already done in hearing aids and CIs. Another approach could be to adapt the frequency focusing of the vocoder to complement the individual’s hearing profile. For example, more bands might be dedicated to higher frequencies for people with a high-frequency hearing loss. Another interesting avenue of investigation might be the design of complementary CI and haptic stimulation strategies. For example, to maximize sound-information transfer, haptic stimulation could focus on providing only lower-frequency sound information and the CI on providing only the higher-frequency information. As has been argued previously^17^, this might reproduce some of the benefits, including those to speech perception, that have been shown for participants who retain low-frequency residual hearing after receiving a CI^54^.

In addition to individualisation of devices and the previously discussed motor placement constraints, there are several other important considerations when developing a device for real-world use. Developers will need to establish the optimal real-time implementation of the tactile vocoder to minimise processing time and power usage (borrowing from current techniques in CIs, which deploy a similar strategy), as well as the utility of methods for reducing the impact of challenges such as wind-noise^6,55^. Other critical work will be required to establish the optimal microphone placement and the ability to stream audio from remote microphones, which has been highly effective for other hearing-assistive devices^56^. As well as these design considerations, it will be important to understand whether tactile speech perception is altered by factors such as skin temperature, which effects tactile sensitivity^57^ and often changes markedly between real-world environments.

The current study showed that formant focusing with the tactile vocoder strategy substantially improves vowel discrimination, without impairing overall consonant discrimination. This strategy is computationally lightweight and can readily be implemented in real time on a compact wearable device to deliver real-world benefit. It could substantially improve outcomes, both for haptic hearing aid users who are unable to access CI technology and for the substantial number of CI users who have impaired vowel perception even in quiet listening conditions.

## Methods

### Participants

Table 1 shows the characteristics of the 20 participants who took part in the study. There were 6 males and 14 females, with an average age of 28 years (ranging from 23 to 36 years). All participants had normal touch perception, as assessed by a health questionnaire and vibro-tactile detection thresholds at the fingertip (see “Procedure”). All the participants reported having no hearing impairment. An inconvenience allowance of £20 was paid to each participant for taking part.

**Table 1:** Participant characteristics. For each participant, the table shows: vibro-tactile detection thresholds measured during screening; wrist temperature measured before testing begun; wrist height, width, and circumference; dominant hand; age; and biological sex.

ID	31.5 Hz thresh. (m/s^−2^)	125 Hz thresh. (m/s^−2^)	Wrist temp. (°C)	Wrist height/ width (mm)	Wrist circum. (mm)	Dom. Hand (L/R)	Age (years)	Sex (M/F)
1	0.021	0.079	31.1	39/58	166	R	36	M
2	0.029	0.101	27.1	34/47	135	R	28	F
3	0.040	0.104	27.2	31/48	139	R	27	F
4	0.026	0.064	32.0	32/47	136	R	25	F
5	0.024	0.181	30.1	36/50	158	R	36	F
6	0.035	0.024	29.5	42/65	186	R	25	M
7	0.045	0.088	31.5	31/44	142	R	31	F
8	0.114	0.240	29.9	40/50	161	R	26	F
9	0.033	0.069	31.0	36/48	149	L	28	F
10	0.039	0.085	29.2	39/49	149	R	30	F
11	0.056	0.088	30.5	39/50	154	R	23	M
12	0.080	0.104	28.4	48/61	188	R	31	M
13	0.031	0.034	32.3	36/43	142	R	25	F
14	0.045	0.048	29.2	36/50	153	R	30	F
15	0.062	0.057	32.1	45/60	190	R	31	M
16	0.049	0.023	31.2	37/49	169	R	27	F
17	0.049	0.091	28.3	35/54	152	R	29	F
18	0.022	0.038	30.3	42/53	170	L	28	M
19	0.082	0.151	29.2	35/46	144	R	23	F
20	0.029	0.075	29.3	39/50	150	R	24	F
Mean	0.046	0.087	30.0	38/51	157	-	28	**-**

### Stimuli

The vibro-tactile stimuli used in the experiment phase (after screening), were generated using the EHS Research Group Phoneme Corpus^18^. This contains an English male and female talker saying each of the 44 British English phonemes, with four recordings of each phoneme per talker.

Table 2 shows the subset of 45 phoneme pairs that were used in the phoneme discrimination task. These were selected to cover a wide range of contrasts while maximizing the functional relevance for potential users of haptic hearing aids. This includes pairs that would not be discriminable using either lip-reading alone or acoustic cues alone with a substantial high-frequency hearing-loss (which is the typical sensorineural hearing-loss profile). Pairs are also included with common vowel and consonant confusions for CI users^26^ and for users of a previous multi-channel tactile aid (the Tactaid-VII)^44^.

**Table 2:** Consonant and vowel pairs used in the experiment, grouped by the type of contrast. Examples of the British English phonemes (bold and underlined) being used in words are also shown (note that these words are for illustration only and were not used in testing).

Consonants		Contrast type	Vowels		Contrast type
*t & p*	*(****t****ea/****p****en)*	*Place in voiceless plosives*	*ɪ & ɑː*	*(k****i****t/c****ar****t)*	*Monophthongs*
*t & k*	*(****t****ea/****k****ey)*	*Place in voiceless plosives*	*iː & æ*	*(s****ea****/b****a****d)*	*Monophthongs*
*k & p*	*(****k****ey/****p****en)*	*Place in voiceless plosives*	*ɔː & ɪ*	*(l****aw****/k****i****t)*	*Monophthongs*
*f &* θ	*(****f****at/pa****th****)*	*Place in voiceless fricatives*	*ʊ & ɑː*	*(p****u****t/c****ar****t)*	*Monophthongs*
*f & s*	*(****f****at/****s****un)*	*Place in voiceless fricatives*	*uː & ʌ*	*(bl****ue****/m****u****d)*	*Monophthongs*
*ʃ & s*	*(****sh****e/****s****un)*	*Place in voiceless fricatives*	*æ & e*	*(b****a****d/b****e****d)*	*Monophthongs*
*d & b*	*(****d****ay/****b****ay)*	*Place in voiced plosives*	*ʊ & ɪ*	*(p****u****t/k****i****t)*	*Monophthongs*
*g & d*	*(****g****et/****d****ay)*	*Place in voiced plosives*	*æ & ɒ*	*(b****a****d/l****o****t)*	*Monophthongs*
*g & b*	*(****g****et/****b****ay)*	*Place in voiced plosives*	*iː & uː*	*(s****ea****/bl****ue****)*	*Monophthongs*
*v & ð*	*(****v****et/****th****is)*	*Place in voiced fricatives*	*ʌ & æ*	*(m****u****d/b****a****d)*	*Monophthongs*
*v & z*	*(****v****et/****z****oo)*	*Place in voiced fricatives*	*uː & ʊ*	*(bl****ue****/p****u****t)*	*Monophthongs*
*ð & z*	*(****th****is/****z****oo)*	*Place in voiced fricatives*	*iː & e*	*(s****ea****/b****e****d)*	*Monophthongs*
*l & r*	*(****l****ot/****r****un)*	*Place in sonorants*	*ɔɪ & eɪ*	*(b****oy****/d****ay****)*	*Diphthongs*
*j & l*	*(****y****et/****l****ot)*	*Place in sonorants*	*ɔɪ & aʊ*	*(b****oy****/n****ow****)*	*Diphthongs*
*m & n*	*(****m****en/****n****ot)*	*Place in sonorants*	*aʊ & eɪ*	*(n****ow****/d****ay****)*	*Diphthongs*
*z & s*	*(****z****ero/****s****un)*	*Voicing*	*ɪə & əʊ*	*(n****ear****/n****o****)*	*Diphthongs*
*ʒ & ʃ*	*(vi****s****ion/****sh****e)*	*Voicing*	*ʊə & eɪ*	*(p****oor****/d****ay****)*	*Diphthongs*
θ *& ð*	*(pa****th****/****th****is)*	*Voicing*	*eə & ʊə*	*(f****air****/p****oor****)*	*Diphthongs*
*t & s*	*(****t****ea/****s****un)*	*Manner*			
*b & w*	*(****b****ay/****w****et)*	*Manner*			
*tʃ & ʃ*	*(****ch****at/****sh****e)*	*Manner*			
*ð & b*	*(****th****is/****b****ay)*	*Manner & place (two-feature)*			
*k & s*	*(****k****ey/****s****un)*	*Manner & place (two-feature)*			
*g & r*	*(****g****et/****r****un)*	*Manner & place (two-feature)*			
*v & s*	*(****v****et/****s****un)*	*Place & voicing (two-feature)*			
θ* & z*	*(pa****th****/****z****ero)*	*Place & voicing (two-feature)*			
*m & v*	*(****m****en/****v****et)*	*Place & voicing (two-feature)*			

The stimulus duration was matched for each phoneme pair by fading out both phonemes with a 20-ms raised-cosine ramp, except for pairs containing a diphthong or containing /g/, /d/, /l/, /r/, /v/, /w/, or /j/*.* For these exceptions, production in isolation (without an adjacent vowel) is impossible or differs acoustically from production in running speech. Duration matching was done to prevent discrimination by comparing the total durations of the stimuli. The start of the stimulus was defined as the first point from the beginning of the sample that the signal reached 1% of its maximum. The fade out reached its zero-amplitude point at the end of the shortest stimulus, which was defined as the first point from the end of the stimulus at which the signal amplitude dropped below 1% of its maximum. The stimuli used in the experiment had a mean duration of 391 ms (ranging from 105 to 849 ms).

In each of the experimental conditions, the audio was converted to vibro-tactile stimulation using a tactile vocoder strategy similar to that used in previous studies^9–11,17–19^. The audio signal intensity was first normalised following ITU P.56 method B^58^. It was then down sampled to a sampling frequency of 16,000 Hz (matching that available in many hearing aids and other compact real-time audio devices). Following this, the signal was passed through a 512^th^-order finite impulse response (FIR) filter bank with eight bands. The frequency limits of these bands differed for the wide, formant, and obstruent focused approaches (see Table 3). With the wide-focused approach, the bands matched those used previously by Fletcher et al.^18^, with the filters equally spaced between 50 and 7000 Hz on the auditory equivalent-rectangular-bandwidth (ERB) scale^59^. With the formant-focused approach, four of the eight bands were spaced between 300 and 1000 Hz (targeting formant 1), three bands were spaced between 1000 and 2500 Hz (targeting formant 2), and one was spaced between 2500 and 7000 Hz (to retain frequency information critical to obstruent phoneme discrimination). With the obstruent-focused approach, one of the eight bands was spaced between 300 and 2500 Hz and the remaining seven were spaced between 2500 and 7000 Hz. This focuses on high-frequency spectral shape information, which is critical to obstruent phoneme perception^25^. Within these frequency ranges, all bands were equally spaced on the ERB scale.

**Table 3:** Lower and upper audio band-pass filter limits for the different tactile vocoder frequency-focusing approaches.

Channel no	Wide focus (low/high in Hz)		Formant focus (low/high in Hz)		Obstruent focus (low/high in Hz)	
1	50	190	300	424	300	2500
2	190	400	424	577	2500	2908
3	400	716	577	767	2908	3376
4	716	1191	767	1000	3376	3914
5	1191	1904	1000	1374	3914	4533
6	1904	2975	1374	1863	4533	5244
7	2975	4584	1863	2500	5244	6061
8	4584	7000	2500	7000	6061	7000

After the band-pass filtering stage, the amplitude envelope was extracted for each band using a Hilbert transform and a zero-phase 6th-order low-pass Butterworth filter, with a corner frequency of 23 Hz (following Fletcher, et al.^18^). These amplitude envelopes were then used to modulate the amplitudes of eight fixed-phase vibro-tactile tonal carriers. The tone frequencies were 94.5, 116.5, 141.5, 170, 202.5, 239, 280.5 and 327.5 Hz. The frequencies were centred on 170 Hz, which is the frequency at which vibration output is maximal for numerous compact haptic actuators. They were spaced based on frequency discrimination thresholds at the dorsal forearm^60^ (as equivalent data is not available at the wrist) and remain within the frequency range (~ 75–350 Hz) that can be reproduced by current commercially available compact, low-powered motors that are suitable for a wrist-worn device.

A frequency-specific gain was applied to each vibro-tactile carrier tone to compensate for differences in vibro-tactile sensitivity across frequency^18,61^. The gains were 13.8, 12.1, 9.9, 6.4, 1.6, 0, 1.7, and 4 dB, respectively. The eight carrier tones were summed together and delivered through vibro-tactile stimulation at a single contact point. The tactile stimuli were scaled to have an equal amplitude in RMS, giving a nominal output level of 1.2 G (141.5 dB ref. 10^–6^ m/s^2^). This intensity can be produced by a range of compact, low-powered haptic actuators. The stimulus level was roved by 3 dB around this nominal level (with a uniform distribution) so that phonemes could not be discriminated using absolute intensity cues. Pink noise was presented through headphones at 60 dBA to ensure audio cues could not be used to discriminate the tactile stimuli.

### Apparatus

Throughout the experiment, participants sat in a vibration isolated, temperature-controlled room (with an average temperature of 23 °C; SD of 0.45 °C). The temperature of the room and of the participant’s skin were measured using a Digitron 2022 T type K thermocouple thermometer. The thermometer was calibrated following ISO 80601-2-56:2017^62^, using the method previously described by Fletcher et al.^18^. Control of skin temperature is important as temperature is known to alter vibro-tactile sensitivity^57^.

During screening, vibro-tactile detection threshold measurements were made using a HVLab Vibro-tactile Perception Meter^63^ with a circular probe that had a 6-mm diameter. The probe gave a constant upward force of 1N and had a rigid surround. A downward force sensor was built into the surround, and the force applied was displayed to the participant. This sensor was calibrated using Adam Equipment OIML calibration weights. The output vibration intensity was calibrated using the Vibro-tactile Perception Meter’s built-in accelerometers (Quartz Shear ICP, model number: 353B43) and a Brüel & Kjær (B&K) Type 4294 calibration exciter. All stimuli had a total harmonic distortion of less than 0.1% and the system conformed to ISO-13091-1:2001^64^.

**Figure 5:**
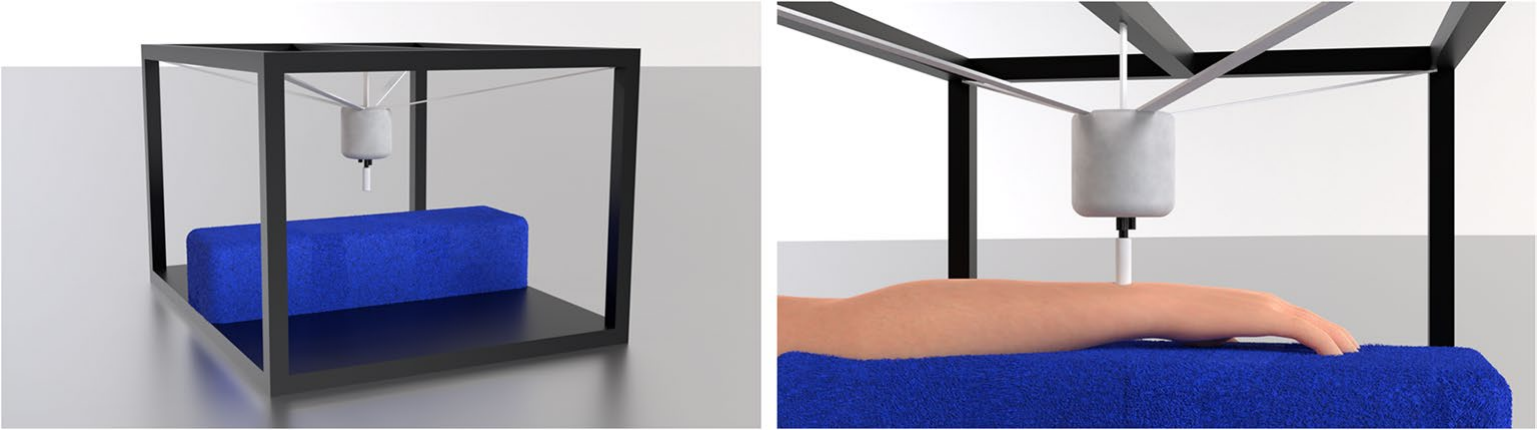
Renders of the EHS Research Group haptic stimulation rig. The left image shows the rig with the participant’s arm not in place. The right image shows a zoomed in view with the participant’s arm resting on the blue foam cushion and the shaker probe contacting the dorsal wrist. Image reproduced from Fletcher et al.^18^ with permission of the authors.

In the experiment phase, the EHS Research Group haptic stimulation rig was used (see Fig. 5)^18^. This consisted of a Ling Dynamic Systems V101 shaker, with a custom-printed circular probe that had a diameter of 10 mm and was made from Verbatim Polylactic Acid (PLA) material. The shaker was driven using a MOTU UltralLite-mk5 sound card, RME QuadMic II preamplifier, and HV Lab Tactile Vibrometer power amplifier. The shaker was suspended using an adjustable elastic cradle from an aluminium strut frame, with the shaker probe pointing downwards (so that it could terminate on the dorsal wrist of the participant). Below the shaker was a foam surface (with a thickness of 95 mm) for the participant’s palmar forearm to rest on. The probe applied a downward force of 1N, which was calibrated using a B&K UA-0247 spring balance. The vibration output was calibrated using a B&K 4533-B-001 accelerometer and a B&K type 4294 calibration exciter. All stimuli had a total harmonic distortion of less than 0.1%.

Masking audio was played from the MOTU UltralLite-mk5 sound card through Sennheiser HDA 300 sound-isolating headphones. The audio was calibrated using a B&K G4 sound level meter, with a B&K 4157 occluded ear coupler (Royston, Hertfordshire, UK). Sound level meter calibration was checked using a B&K Type 4231 calibrator.

### Procedure

For each participant, the experiment was completed in one session that lasted approximately two hours. Participants gave informed consent to take part and completed a screening questionnaire. This ensured that they (1) did not suffer from conditions that could affect their sense of touch, (2) had not had any injury or surgery on their hands or arms, and (3) had not been exposed to intense or prolonged hand or arm vibration in the previous 24 h. The participant’s skin temperature was then measured on the index fingertip of the dominant arm. Participants were only allowed to continue when their skin temperature was between 27 and 35 °C.

Next, vibro-tactile detection thresholds were measured at the index fingertip following BS ISO 13091-1:2001^64^. During the threshold measurements, participants applied a downward force of 2N (monitored using the HVLab Vibro-tactile Perception Meter display). Participants were required to have touch perception thresholds in the normal range (< 0.4 m/s^−2^ RMS at 31.5 Hz and < 0.7 m/s^−2^ RMS at 125 Hz), conforming to BS ISO 13091‑2:2021^65^. The fingertip was used because normative data was not available for the wrist. If participants passed the screening phases, the dimensions of the wrist were measured at the point where the participant would usually wear a wristwatch, and they then progressed to the experiment phase.

In the experiment phase, participants sat in front of the EHS Research Group haptic stimulation rig (Fig. 5), with the forearm of their dominant arm resting on a foam surface. The probe from the shaker was adjusted so that it contacted the centre of the dorsal wrist (at the position where the participant would normally wear a wristwatch). The participant’s skin temperature was required to be between 27 and 35 °C before testing began.

The experiment phase involved a previously developed three-interval, three-alternative forced-choice phoneme discrimination task^18^. For each trial, one phoneme pair from either the male or female talker was used (see “Stimulus”). Two intervals contained one phoneme from the pair (randomly selected) and one interval contained the other phoneme from the pair. The intervals were separated by a gap of 250 ms and the order of the intervals was randomised. The participant’s task was to select which of the three intervals contained the oddball stimulus (i.e., the phoneme presented only once) via a key press. They were instructed to ignore the overall intensity of the vibration in each interval (as the level roving that was deployed to prevent the use of overall intensity for discrimination rendered this an unreliable cue). Visual feedback, which indicated whether the response was correct or incorrect, was displayed for 500 ms after each trial.

The percentage of phonemes correctly discriminated was measured for three conditions, each with a different band-pass filter allocation (Table 3). For each condition, all the phoneme pairs were tested (Table 2) with both the male and female talker. For each talker, each phoneme pair was measured four times, with the phoneme sample randomly selected in each trial from the four samples available in the corpus. This meant that there were a total of 1080 trials for each participant. All phoneme pairs and conditions were measured for each repeat in sequence, with the order of trials randomised each time.

The experimental protocol was approved by the University of Southampton Faculty of Engineering and Physical Sciences Ethics Committee (ERGO ID: 68477). All research was performed in accordance with the relevant guidelines and regulations.

### Statistics

The percentage of correctly discriminated phonemes was calculated for each condition for the male and female talker. Primary analysis consisted of a three-way RM-ANOVA, with the factors ‘Focusing approach’ (wide, formant, or obstruent), ‘Phoneme type’ (consonant or vowel), and ‘Talker’ (male or female). Contrasts were also run to compare performance for the obstruent and formant focused approaches to the baseline wide-focused approach. Data were determined to be normally distributed based on visual inspection, Kolmogorov–Smirnov, and Shapiro–Wilk tests. Mauchly’s test indicated that the assumption of sphericity had not been violated. The RM-ANOVA used an alpha level of 0.05.

Planned* post-hoc* analyses were then conducted. These assessed whether the effect of formant and obstruent focusing (compared to the baseline wide focusing) differed across all phonemes or for consonants or vowels alone. A Bonferroni-Holm correction^66^ for multiple comparisons applied was applied (3 comparisons in total).

A second set of unplanned two-tailed *t*-tests were also conducted. These assessed the differences between the baseline (wide focusing) and either the formant focused or obstruent focused conditions for each phoneme subgroup (see Table 2). A Bonferroni-Holm correction for multiple comparisons was applied (25 comparisons in total).

Finally, six Pearson’s correlations were run between either participant age or detection thresholds for a 125 Hz vibro-tactile tone (measured during screening) and the overall phoneme discrimination scores with either the wide focused, formant focused, or obstruent focused approach. These exploratory additional analyses were not corrected for multiple comparisons, as it was hypothesised that no correlation would be found in any of these conditions, following results from previous studies (e.g.^18^).

## Data Availability

The datasets generated and analysed during the current study are available in the University of Southampton’s Research Data Management Repository at: 10.5258/SOTON/D2969.
